# Experimental peripheral arterial disease: new insights into muscle glucose uptake, macrophage, and T‐cell polarization during early and late stages

**DOI:** 10.1002/phy2.234

**Published:** 2014-02-25

**Authors:** Maxime Pellegrin, Karima Bouzourène, Carole Poitry‐Yamate, Vladimir Mlynarik, François Feihl, Jean‐François Aubert, Rolf Gruetter, Lucia Mazzolai

**Affiliations:** 1 Division of Angiology, University Hospital of Lausanne, Lausanne, Switzerland; 2 Centre d'Imagerie Biomédicale, Ecole Polytechnique Fédérale de Lausanne, Lausanne, Switzerland; 3 Division of Clinical Pathophysiology, University Hospital of Lausanne, Lausanne, Switzerland

**Keywords:** M1 and M2 macrophages, MR spectroscopy, peripheral arterial disease, Th1 and Th2 cells, tomography

## Abstract

Peripheral arterial disease (PAD) is a common disease with increasing prevalence, presenting with impaired walking ability affecting patient's quality of life. PAD epidemiology is known, however, mechanisms underlying functional muscle impairment remain unclear. Using a mouse PAD model, aim of this study was to assess muscle adaptive responses during early (1 week) and late (5 weeks) disease stages. Unilateral hindlimb ischemia was induced in ApoE^−/−^ mice by iliac artery ligation. Ischemic limb perfusion and oxygenation (Laser Doppler imaging, transcutaneous oxygen pressure assessments) significantly decreased during early and late stage compared to pre‐ischemia, however, values were significantly higher during late versus early phase. Number of arterioles and arteriogenesis‐linked gene expression increased at later stage. Walking ability, evaluated by forced and voluntary walking tests, remained significantly decreased both at early and late phase without any significant improvement. Muscle glucose uptake ([18F]fluorodeoxyglucose positron emission tomography) significantly increased during early ischemia decreasing at later stage. Gene expression analysis showed significant shift in muscle M1/M2 macrophages and Th1/Th2 T cells balance toward pro‐inflammatory phenotype during early ischemia; later, inflammatory state returned to neutrality. Muscular M1/M2 shift inhibition by a statin prevented impaired walking ability in early ischemia. High‐energy phosphate metabolism remained unchanged (31‐Phosphorus magnetic resonance spectroscopy). Results show that rapid transient muscular inflammation contributes to impaired walking capacity while increased glucose uptake may be a compensatory mechanisms preserving immediate limb viability during early ischemia in a mouse PAD model. With time, increased ischemic limb perfusion and oxygenation assure muscle viability although not sufficiently to improve walking impairment. Subsequent decreased muscle glucose uptake may partly contribute to chronic walking impairment. Early inflammation inhibition and/or late muscle glucose impairment prevention are promising strategies for PAD management.

## Abstract (precis)

e00234

Mechanisms responsible for functional muscle impairment in peripheral artery disease patients remain unknown. Our results show that both pro‐inflammatory T cells and macrophages are implicated in early peripheral ischemia whereas late ischemia is associated with impaired muscle glucose uptake in a mouse model of peripheral arterial disease (PAD).

## Introduction

Peripheral arterial disease (PAD) is a common disorder mainly due to atherosclerosis characterized by stenosis and/or obstruction of lower limbs arteries leading to decreased muscle perfusion and oxygenation. PAD represents a major public health issue. Its prevalence is ~12% in the adult population, increasing to 20% above 70 years (Hirsch et al. 2006; Norgren et al. 2007; Olin et al. 2010). Symptomatic PAD patients suffer symptoms of intermittent claudication (IC), defined as fatigue, discomfort, or pain occurring in limb muscles during effort, due to exercise‐induced ischemia, with rapid relief at rest (Hirsch et al. 2006). As a result, patients with PAD and IC are physically impaired and have a markedly reduced quality of life (Hirsch et al. 2006; Norgren et al. 2007; Olin et al. 2010). Moreover, PAD is associated with a significant increase in cardiovascular (CV) morbidity (myocardial infarction and stroke) and mortality (CV and all cause) (Hirsch et al. 2006; Norgren et al. 2007; Olin et al. 2010). PAD management includes strict CV risk factors control, and patient encouragement to regular walking exercise. If needed, revascularization procedures are proposed to avoid lower limb amputation. Unfortunately, no specific treatment for PAD is yet available.

Due to the complexity and multifactorial origins of PAD, as well as the differences in muscular adaptive responses, precise PAD pathophysiological mechanisms are still largely unknown. Although blood flow limitation to active muscle is of critical importance, little is known about factors independent of blood flow and intrinsic to skeletal muscle that may also contribute to disease process and functional limitations in PAD patients.

More than 90% of cases of PAD are secondary to atherosclerosis, which is now recognized as a chronic inflammatory disease. Atherosclerotic plaques contain abundant immune cells, mainly macrophages and CD4^+^ T cells, that orchestrate many of the inflammatory processes occurring throughout atherogenesis (Hansson and Hermansson 2011; Ketelhuth and Hansson 2011). These cells can polarize toward different phenotypes (pro‐inflammatory or anti‐inflammatory) according to various stimuli present in their surrounding microenvironment. Thus, CD4^+^ T‐cell subtype Th1 (pro‐inflammatory cells) and CD4^+^ T‐cell subtype Th2 (anti‐inflammatory cells) exist in plaques, each having a distinct function influencing lesion's fate, that is, development of rupture‐prone unstable versus stable plaque phenotype (Hansson and Hermansson 2011; Ketelhuth and Hansson 2011). Likewise, macrophages can polarize into two different subsets: classically pro‐inflammatory M1 macrophages, driven by Th1 cytokines, or alternatively anti‐inflammatory M2 macrophages, driven by Th2 cytokines. Recent evidence indicates that macrophage polarization balance is a crucial element in determining plaque outcome (Hoeksema et al. 2012). Besides their role in atherosclerosis, macrophages and CD4^+^ T cells have been implicated in ischemia‐induced neovascularization through the synthesis of local angiogenic/arteriogenic factors (Silvestre et al. 2008). However, although emerging evidence shows a role for CD4^+^ T cells and macrophage phenotype switch in atherosclerosis, no study has addressed this significance in PAD.

Few studies have reported abnormal skeletal muscle metabolism in patients with PAD and IC, including impaired skeletal muscle glucose uptake (Pipinos et al. 2007, 2008; Anderson et al. 2009; Pande et al. 2011), however, this potential mechanistic explanation has not been studied in early and late phases of PAD.

Using a mouse model of peripheral ischemia with impaired walking ability, aim of present study was to assess skeletal muscle adaptive responses during early and late stages of PAD focusing on glucose and high‐energy phosphates metabolism, and M1/M2 macrophages and Th1/Th2 cells polarization.

## Methods

### Mouse model of PAD and IC

Unilateral hindlimb ischemia was induced in 14–16‐week old male hypercholesterolemic and atherosclerotic C57BL/6J Apolipoprotein E knock‐out (ApoE^−/−^) mice (Charles River Laboratories, L'Arbresle Cedex, France) by right common iliac artery ligation. Briefly, mice were anesthetized using isoflurane inhalation (1–2% in O_2_) and placed on a heated pad during surgery. Hindlimbs and inferior abdominal area were shaved. Through a small abdominal incision, right common iliac artery was exposed and ligated with 7–0 silk suture just above the internal–external iliac artery bifurcation. Iliac vein and nerve were preserved. Abdominal incision was then sutured with a resorbable 5–0 silk suture. Sham‐operated contralateral nonischemic hindlimb served as control. One week prior to surgery, mice were treated with Dafalgan (200 mg/kg) via the drinking water for 14 days. In addition, mice were administered Temgesic (0.01 mg/kg, s.c.) following surgery.

Mice were fed regular rodent chow, and accessed water ad libitum throughout the study. Animal experiments were performed according to the Swiss Federal guidelines (Ethical Principles and Guidelines for Experiments on Animals). The protocol was approved by the local Institutional Animal Committee (Service Vétérinaire Cantonal, Lausanne, Switzerland). All efforts were made to minimize animal suffering during the experiments.

### In vivo transcutaneous oxygen pressure measurement

Before ischemia, 1 week, and 5 weeks postischemia, skin oxygenation in ischemic hindlimb was determined by measuring transcutaneous oxygen pressure (TcPO_2_) using a TcPO_2_‐monitoring system equipped with a Clark electrode (TCM30; Radiometer, Copenhagen, Denmark). TcPO_2_ measurements are routinely used in vascular clinical practice as a measure of ischemia severity in lower extremities of PAD patients. Reproducibility and accuracy of TcPO_2_ measurements in mice were tested in preliminary experiments in control nonischemic mice. Measurements were performed in anesthetized mice placed in the supine position on a heated pad to maintain body temperature at 37 ± 1°C. Prior to each measurement, electrode calibration was performed according to manufacturer's instructions. After calibration, the electrode was connected to a ring filled with contact solution, to avoid oxygen air interference, and fixed to the skin just above the knee. After a 15 min period of stabilization, TcPO_2_ (expressed in mmHg) was continuously recorded during 15 min. Values measured at 15 min were used for analysis.

### In vivo laser Doppler perfusion imaging

Skin perfusion of both ischemic and contralateral nonischemic hindlimbs was evaluated using a Laser Doppler Imager (Moor Instruments, Axminster, U.K.) in anesthetized mice placed in a prone position on a heating pad. Before ischemia, 1 week, and 5 weeks postischemia, five consecutive plantar foot images were recorded at 30‐sec intervals, and averaged. Perfusion status was calculated on the basis of colored histogram pixels within the region of interest (ROI) using Moor LDI Image Review software. Tissue perfusion in ischemic hindlimb was expressed as a percentage of that measured in contralateral nonischemic hindlimb. This calculation allows minimizing biases due to variables such as ambient light and even minimal temperature variations.

### Total walking distance assessment

Total 24‐h walking distance (24hTWD) was assessed at three time points: before ischemia, 1 week, and 5 weeks postischemia. Mice were housed in individual cages containing a 12‐cm diameter wheel and were free to run during 24 h. The wheel was connected to a counter recording number of revolutions allowing 24hTWD (kilometers) calculation for each animal.

### Maximal walking distance and time assessment

Maximal walking distance (MWD) and maximal walking time (MWT) were determined before ischemia, 1 week, and 5 weeks postischemia using a forced treadmill (Columbus Instruments, Columbus, OH). Mice were subjected to an incremental speed protocol, starting at a speed of 9 m/min for 3 min with an increase of 2 m/min every 3 min until speed reached 19 m/min (0% slope). Mice were encouraged to run as long as possible with the use of an electric grid located at the back of the treadmill (1.5 mA, 3 Hz). The test was stopped when mice were exhausted (remained on the shock grid for five continuous seconds). MWD (kilometers) and time (minutes) were then calculated for each animal.

### Clinical evaluation of ischemia and limb function

At 1 and 5 weeks postischemia, mice were observed and scored according to an ischemia grade scale (0 = normal, 1 = foot discoloration, and 2 = tissue necrosis). Limb function was also assessed using a gait abnormality grade scale (0 = normal, 1 = limping, 2 = dragging of foot).

### In vivo [^18^F]fluorodeoxyglucose PET imaging and glucose metabolism of hindlimb muscle

At 1 and 5 weeks postischemia, noninvasive [^18^F]fluorodeoxyglucose (^18^FDG) positron emission tomography (PET) hindlimbs imaging was performed using an avalanche photodiode microPET scanner (LabPET4; Gamma Medica, Sherbrooke, Canada) (Seyer et al. 2013). Mice were anesthetized with a mixture of 1.5% isoflurane in 100% O_2_ (0.9 L/mL, 2.5 bars) and tail vein catheterized into. Animals were prone positioned with extended legs. Fifty‐minute list mode acquisitions were acquired with field of view (FOV) containing both ischemic and contralateral nonischemic hindlimbs. i.v. injection of ^18^FDG (≈50 MBq) through tail vein catheter was initiated within the first 10 sec of PET scan, followed by 100–500 *μ*L of saline chase solution. Number of detected single events/s was used to evaluate and control intravenous ^18^FDG delivery. During the entire scanning period, mice were maintained under isoflurane anesthesia using a face mask. Temperature and breathing rate were continuously monitored. An energy window of 250–650 keV and a coincidence timing window of 22.2 nanoseconds were used. For image reconstruction, storage of coincidence events, recorded in list mode files during the PET scan, were binned according to their line of response, as previously described (Selivanov et al. 2000). Voxel size measured 0.5 × 0.5 × 1.2 mm, giving a typical resolution of 1.2 mm at the center of FOV. For spatial histogramming, scans of 50 min duration were reconstructed in three blocks (15, 15 and 20 min, respectively) using a FOV of 46 mm, a span field of 31 and a “maximum likelihood expectation maximum” (MLEM) from 20 to 60 iterations, intermediate images were saved every five iterations. After correcting for different count‐rates of each line of response and for quantitative ^18^FDG calibration, images of accumulated intracellular ^18^FDG‐6P at steady state were quantitatively expressed using standardized uptake value (SUV) (mean ROI activity [kBq/cm^3^])/(injected dose [kBq]/body weight [g]). Images were corrected for nonuniformity of the scanner response, dead time count losses, and physical decay from time of injection. No correction was applied for attenuation and partial‐volume effects. Images were analyzed with PMOD 3.2 software (PMOD Technologies, Zurich, Switzerland). ROIs were manually drawn by optical reading of well‐delineated hindlimb muscle. Glucose uptake in ischemic hindlimb muscle was expressed as percentage of that in contralateral nonischemic hindlimb.

Inter‐hindlimb variability in individual mice was assessed in three control nonischemic animals. Results demonstrated <2% variability in ^18^FDG uptake between right and left hindlimb.

### In vivo 31‐phosphorus magnetic resonance spectroscopy

Mice were measured on a 9.4 T Varian VNMRS spectrometer (Varian, Palo Alto, CA) in supine position 1 and 5 weeks postischemia. Animal anesthesia, body temperature, and breathing rate were continuously monitored through the measurement. A home‐built 18 mm‐diameter dual ^1^H quadrature/10 mm‐diameter ^31^P single‐loop surface radiofrequency coil was used and positioned over mice hindlimbs. Thereafter, hindlimbs were fixed in order to prevent any movement leading to signal deterioration. T2‐weighted turbo‐spin‐echo images were obtained in the axial plane of ischemic and nonischemic quadriceps muscles using a FOV 30 × 30 mm and 1 mm slice thickness. For spectroscopy, volume of interest (VOIs) of about 60 mm^3^ was chosen. Static field homogeneity in selected VOI was adjusted by an echo‐planar‐imaging version of FASTMAP (fast, automatic shimming technique by mapping along projections) using the 1H signal of water (Gruetter and Tkac 2000). Spectroscopic localization was achieved by outer volume saturation, that is, by applying slice selective inversion in the upper horizontal plane and saturation pulses in all planes around the selected VOI (Mlynarik et al. 2006). Overall, 160 transients were collected with a repetition time of 4 sec. Total measurement time for imaging and ^31^P spectroscopy was about 1 h. Peak intensities of inorganic phosphate (Pi), phosphocreatine (PCr), and adenosine triphosphate (*γ*‐ATP) were obtained by fitting to a Lorentzian function using AMARES (Vanhamme et al. 1997) from the jMrui software (http://sermn02.uab.cat/mrui7). Ratios of PCr to *γ*‐ATP and PCr to Pi were calculated from the respective peaks intensities. PCr/*γ*‐ATP and PCr/Pi in ischemic hindlimb were expressed as percentage of PCr/*γ*‐ATP and PCr/Pi in nonischemic contralateral hindlimb, respectively. Preliminary data showed similar PCr to *γ*‐ATP and PCr to Pi ratios between right and left hindlimb in control nonischemic mice.

### Muscle histology and immunohistochemistry analysis

On week 5 postischemia, ischemic quadriceps and gastrocnemius skeletal muscles were isolated and fixed with 10% buffered formalin. Quadriceps and gastrocnemius muscles were also harvested from two independent groups of mice sacrificed at pre‐ischemia (control nonischemic mice), and 1 week postischemia. After fixation, specimens were further embedded in paraffin, and tissue sections (5 *μ*m thick) prepared. Transverse sections were hematoxylin and eosin stained. Pictures were acquired with a high‐sensitivity color camera (Leica DC300F Camera, Wetzler, Germany). Muscle fiber size (*μ*m^2^) was determined using morphometric analysis (Qwin software, Leica). For each sample, a minimum of 50 muscle fiber sizes were quantified, and results averaged.

For arteriogenesis evaluation, muscle sections were immunostained with a mouse monoclonal *α*‐SM actin antibody, followed by a secondary biotinylated anti‐mouse antibody. Antibodies were revealed with a peroxidase‐linked avidin‐biotin detection system (Vectastain ABC kit; Vector Laboratories, Burlingame, CA) as previously described (Mazzolai et al. 2004). The number of arterioles in each section was counted in a blinded fashion in five randomly selected fields using the Qwin software. Arteriolar density, number of arterioles per muscle fiber, was then calculated.

### Real‐time reverse transcription‐polymerase chain reaction

Total RNA was isolated from ischemic and nonischemic quadriceps and gastrocnemius muscles, at both 1 and 5 weeks postischemia, using Trizol reagent (Invitrogen, Switzerland) followed by the RNeasy Cleanup Kit (Qiagen, Switzerland). RNA concentration and purity were spectrophotometrically estimated by calculating the A_260_/A_280_ ratio. cDNA was then synthesized by reverse transcription using the iScriptT™ cDNA Synthesis Kit from Bio‐Rad (Reinach, Switzerland). Quantitative Real time PCR was performed on IQ™‐Cycler (Bio‐rad, Switzerland) using iQ^™^ SYBR^®^ Green Supermix (Bio‐Rad, Switzerland) according to manufacturer's protocols. The following primers were used: Hypoxia‐inducible factor‐1*α* (HIF‐1*α*): sens 5′‐TCAAGTCAGCAACGTGGAAG‐3′, and antisense 5′‐ TATCGAGGCTGTGTCGACTG‐3′; Angiopoietin‐2 (ANG2): sens 5′‐GCATGTGGTCCTTCCAACTT‐3′, and antisens 5′‐ TGGTGTCTC TCAGTGCCTTG‐3′; CD11c: sens 5′‐ACACAGTGTGCTCCAGTATGA‐3′, and antisense 5′‐GCCCAGGGATATGTTCACAGC; CD206: sense 5′‐CATGGATGTTGATGGCTACTGGAG‐3′, and antisense 5′‐GTCTGTTCTGACTCTGGACACTTG‐3′; Interferon‐gamma (IFN‐*γ*): sense 5′‐ TGAGACAATGAACGCTACACACTG‐3′, and antisense 5′‐TTCCACATCTATGCCACTTGAG‐3′; Interleukin‐4 (IL‐4): sense 5′‐TCAACCCCCAGCTAGTTGTC‐3′, and antisense: 5′‐TGTTCTTCGTTGCTGTGAGG‐3′; and 36B4: sense 5′‐ATGGGTACAAGCGCGTCCTG‐3′, and antisense 5′GCCTTGACCTTTTCAGTAAG‐3′. All samples were run in duplicates. Post PCR melting curves were analyzed to ensure primer specificity. Data were analyzed using the comparative threshold cycles (CT) method (Livak and Schmittgen 2001). Briefly, all results were normalized for the housekeeping 36B4 gene. mRNA expression of genes from ischemic muscles was expressed as fold change in those from non ischemic contralateral muscles.

### Statin treatment

ApoE^−/−^ mice were administrated oral atorvastatin (20 mg/kg per day in drinking water, kindly provided by Pfizer) (Wang et al. 2011) 1 day before ischemia until 1 week postischemia. Nontreated mice were used as controls. 24hTWD was assessed in mice before and after atorvastatin treatment using the voluntary walking test while gene expression analysis for CD11c and CD206 was performed in ischemic and nonischemic quadriceps at the end of the treatment according to methods described above.

### Statistical analysis

All data are expressed as mean ± SD. Statistical significance was evaluated using one‐way analysis of variance (ANOVA) or repeated measures ANOVA followed by the Tukey post hoc analysis for multiple comparisons. For comparison between two groups, statistical significance was determined by the paired or unpaired *t*‐test. Differences in ischemia and limb function scores were evaluated by the Fisher exact test. A value of *P* < 0.05 was considered to be statistically significant.

## Results

### Mouse model of PAD

**Figure 1.**
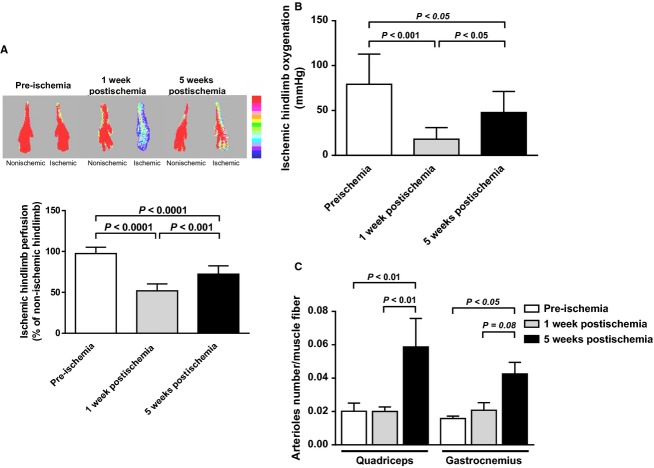
In vivo hindlimb tissue perfusion and oxygenation, and muscles arteriolar density before ischemia, at early, and later stages. (A) Upper, Representative laser Doppler images of ischemic (right) and contralateral nonischemic (left) paws at the various time points. Low perfusion is indicated by blue color while high perfusion by the red color according to a color scale. Lower, Quantification of ischemic hindlimb tissue perfusion expressed as percentage of nonischemic hindlimb perfusion (*n* = 7–10 animals per time point). (B) Quantification of ischemic hindlimb tissue oxygenation using TcPO_2_ measurement (*n* = 10 animals per time point). Results are reported in mmHg. (C) Quantification of arteriolar density in ischemic quadriceps (proximal to ischemia) and gastrocnemius (distal to ischemia) muscles, measured as the number of *α*‐SM actin positive arterioles per muscle fiber (*n* = 4–7 animals per time point).

Following artery ligation, perfusion of ischemic hindlimbs, assessed by laser Doppler, significantly decreased by 47% 1 week postintervention (early stage of ischemia) compared to the pre‐ischemic situation (*P* < 0.0001; Fig. 1A). Perfusion remained significantly low (−26%) also at later phase of ischemia (5 weeks postartery ligation; *P* < 0.0001 vs. pre‐ischemia) although values resulted higher than those observed in the earlier ischemic phase (*P* < 0.001; Fig. 1A). Similarly, tissue oxygenation of ischemic hindlimbs, assessed by TcPO_2_, significantly decreased 1 week postischemia compared to the pre‐ischemic situation (*P* < 0.001; Fig. 1B). This decrease remained significant at 5 weeks (*P* < 0.05) although values were increased compared to the 1 week levels (*P* < 0.05; Fig. 1B).

Number of ischemic muscles arterioles was also evaluated using *α*‐SM actin immunostaining. Consistent with laser Doppler imaging and TcPO_2_ results, number of arterioles significantly increased, after 5 weeks, in ischemic quadriceps muscle (*P* < 0.01 vs. 1 week; Fig. 1C). At similar time point, number of arterioles increased also in gastrocnemius muscle although not significantly (*P* = 0.08; Fig. 1C).

Along the same line, mRNA expression of pro‐angiogenic factor HIF‐1*α* was significantly upregulated both in ischemic quadriceps (2.2‐fold) and gastrocnemius (1.5‐fold) muscles already at 1 week postischemia (*P* < 0.05 vs. respective nonischemic muscle; Table 1). ANG2 expression (an arteriogenic factor) was significantly upregulated in ischemic quadriceps muscle at 5 weeks postischemia (1.3‐fold, *P* < 0.001 vs. nonischemic one), and in ischemic gastrocnemius muscles both at 1 and 5 weeks postischemia (2.1‐fold and 1.7‐fold, respectively, *P* < 0.05 vs. nonischemic muscles) (Table 1).

**Table 1.** Proangiogenic/arteriogenic gene expression in quadriceps and gastrocnemius muscles at early and later stages of peripheral ischemia.

	1 week postischemia				5 week postischemia			
	Quadriceps muscle		Gastrocnemius muscle		Quadriceps muscle		Gastrocnemius muscle	
	Nonischemic hindlimb	Ischemic hindlimb	Nonischemic hindlimb	Ischemic hindlimb	Nonischemic hindlimb	Ischemic hindlimb	Nonischemic hindlimb	Ischemic hindlimb
HIF‐1*α*	1 ± 0.21	2.22 ± 0.21*	1 ± 0.17	1.54 ± 0.28*	1 ± 0.13	1.09 ± 0.18†	1 ± 0.24	1.13 ± 0.30
ANG2	1 ± 0.27	1.04 ± 0.44	1 ± 0.20	2.06 ± 0.20***	1 ± 0.10	1.33 ± 0.15*†	1 ± 0.21	1.68 ± 0.20*

Results are expressed as fold change in expression over respective contralateral nonischemic muscles, set at 1 (*n* = 8–10 animals per time point).

*P < 0.05, ***P < 0.001 versus respective nonischemic muscle.

^†^*P* < 0.05 versus 1 week postischemia.

### Walking abilities of mice at early and late stages of limb ischemia

**Figure 2.**
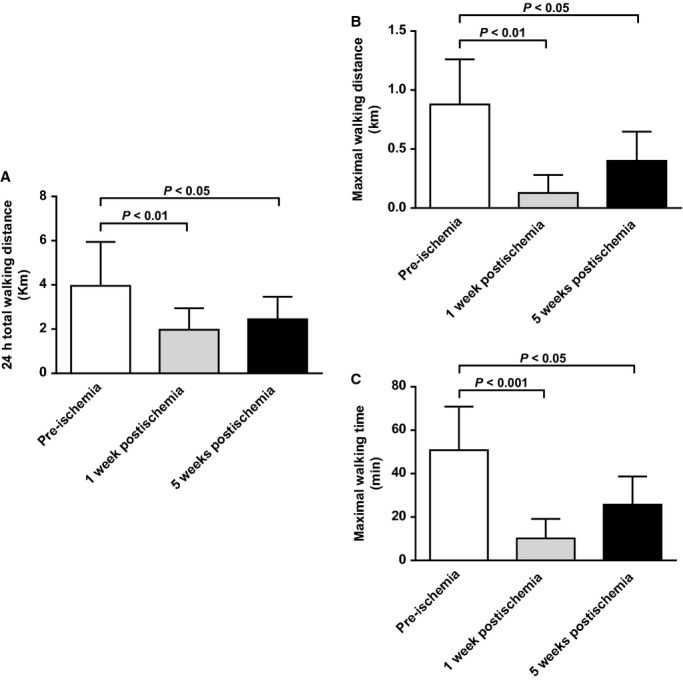
Walking ability of mice before ischemia, at early, and later stages. (A) Quantification of 24 h total walking distance (24hTWD) using a 24 h voluntary running wheel test (*n* = 9 animals per time point). (B) Quantification of maximal walking distance (MWD), and (C) Quantification of maximal walking time (MWT) as measured by forced incremental treadmill running test (*n* = 5–7 animals per time point). Reported results are expressed in kilometers or minutes.

Walking ability of mice was evaluated at early (1 week postartery ligation) and late (5 weeks postartery ligation) stages of ischemia using voluntary and forced walking tests. 24hTWD significantly decreased by 50% during early stages of ischemia compared to the pre‐ischemic phase (*P* < 0.01; Fig. 2A). Walking impairment significantly persisted at 5 weeks (*P* < 0.05 vs. pre‐ischemia; Fig. 2A) and up to 14 weeks postischemia (data not shown). Similarly, both MWD and MWT significantly decreased by 85% (*P* < 0.01 vs. pre‐ischemia), and 80% (*P* < 0.001 vs. pre‐ischemia), respectively during early ischemic phase (Fig. 2B and C). These decreases remained significant also after 5 weeks (*P* < 0.05 vs. pre‐ischemia; Fig. 2B and C). Interestingly, no significant improvement in walking ability was observed between early and late phases of ischemia (Fig. 2A–C).

**Figure 3.**
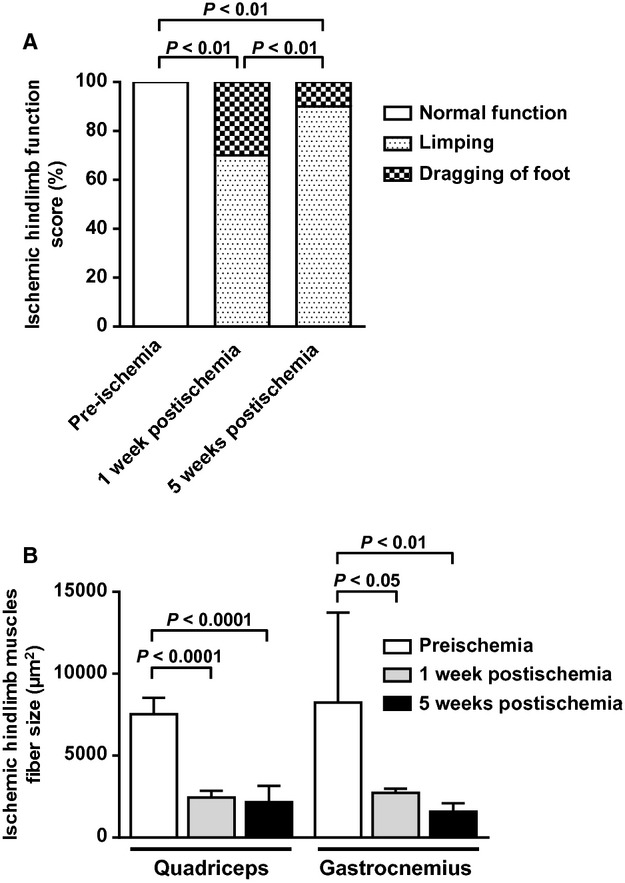
Assessment of limb function, and histological assessment of limb muscular atrophy before ischemia, at early, and later stages. (A) Quantification of limb function score. Data represent percentage of mice presenting the analyzed characteristic (*n* = 10 animals per time point). (B) Quantification of muscle fiber area in cross sections of quadriceps and gastrocnemius muscles (*n* = 4–7 animals per time point; hematoxylin and eosin staining.

Clinical observation of mice revealed impaired limb function both at early and late stages of ischemia, characterized by limping and foot dragging (Fig. 3A). No discoloration and/or necrosis was observed. As expected, histological analysis showed muscle fiber atrophy in ischemic quadriceps and gastrocnemius muscles (Fig. 3B).

### In vivo resting muscle glucose metabolism in early and late stages of limb ischemia

**Figure 4.**
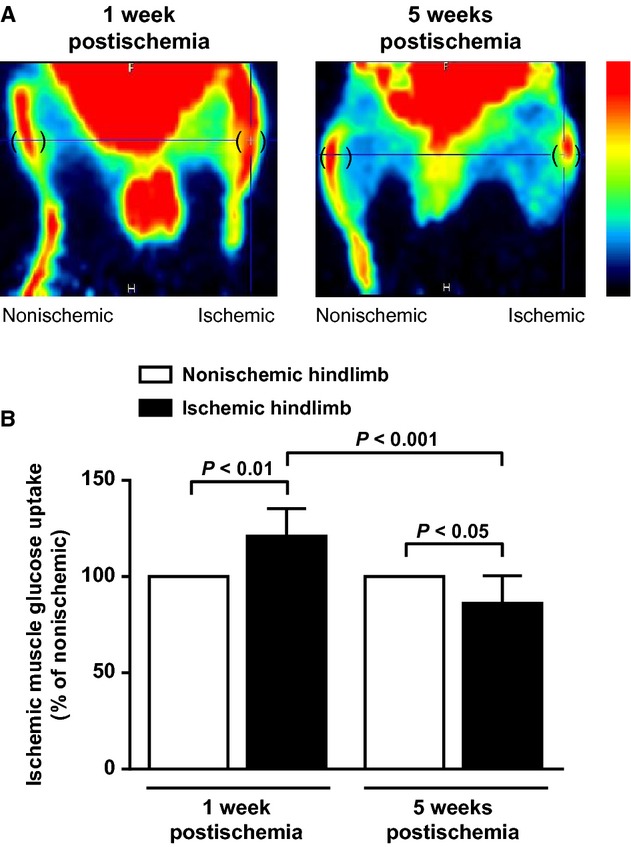
In vivo hindlimb muscle glucose uptake at early and later stages of peripheral ischemia. (A) Representative images of ^18^FDG PET images of phosphorylated ^18^FDG levels at steady‐state in ischemic (right) and nonischemic contralateral (left) hindlimbs. Round brackets indicate regions of interest over gastrocnemius hindlimb muscles. A color scale illustrates glucose uptake variations from minimal (black) to maximal (red) values. (B) Quantification of glucose uptake in resting ischemic hindlimb gastrocnemius muscle. Results are expressed as percentage of glucose uptake in nonischemic hindlimb muscle, set at 100% (*n* = 7 animals per time point).

Noninvasive [^18^F]fluorodeoxyglucose PET imaging was used to quantify glucose uptake in ischemic hindlimb muscles. Representative ^18^FDG PET images of phosphorylated ^18^FDG levels at steady state are shown in Figure 4A. During early phase, glucose uptake significantly increased in ischemic hindlimb muscle compared to the nonischemic one (*P* < 0.01; Fig. 4A and B). On the contrary, at later stages of ischemia, ischemic muscle glucose uptake significantly decreased not only compared to the nonischemic muscle (*P* < 0.05) but also compared to the early stage within the ischemic muscle (*P* < 0.001) (Fig. 4A and B).

### In vivo resting muscles energetic state in early and late stages of limb ischemia

**Figure 5.**
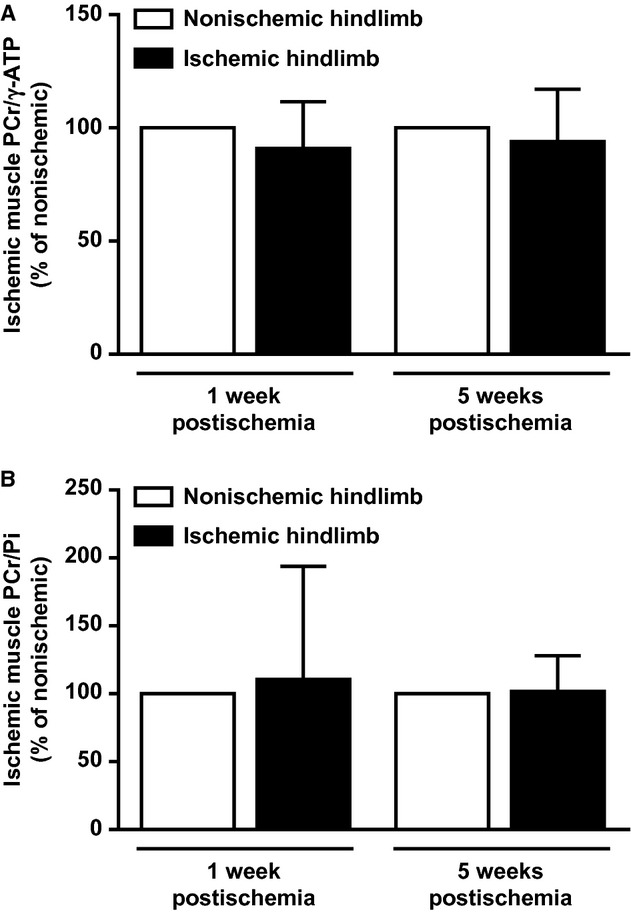
In vivo hindlimb muscle bioenergetics at early and later stages of peripheral ischemia. (A) Ratio of PCr to *γ*‐ATP and (B) Ratio of PCr to Pi in resting ischemic hindlimb muscle, as measured by ^31^P‐MRS. Results are expressed as percentage of nonischemic contralateral hindlimb muscle, set at 100% (*n* = 5–8 animals per time point).

Muscle energy state was assessed by ^31^P‐MRS. Peaks of high‐energy phosphate metabolites (PCr, *γ*‐ATP), and Pi were, respectively, identified in spectra measured from ischemic and nonischemic contralateral hindlimb muscle. As shown in Figure 5A and B, PCr/*γ*‐ATP and PCr/Pi ratios were not different between ischemic and nonischemic hindlimb muscles either at early or at later stages of ischemia. Similarly, no change in integral intensity of the ATP peaks (in terms of signal‐to‐noise ratio) was observed in ischemic hindlimbs.

### Muscle macrophage phenotype in early and late stages of limb ischemia

**Figure 6.**
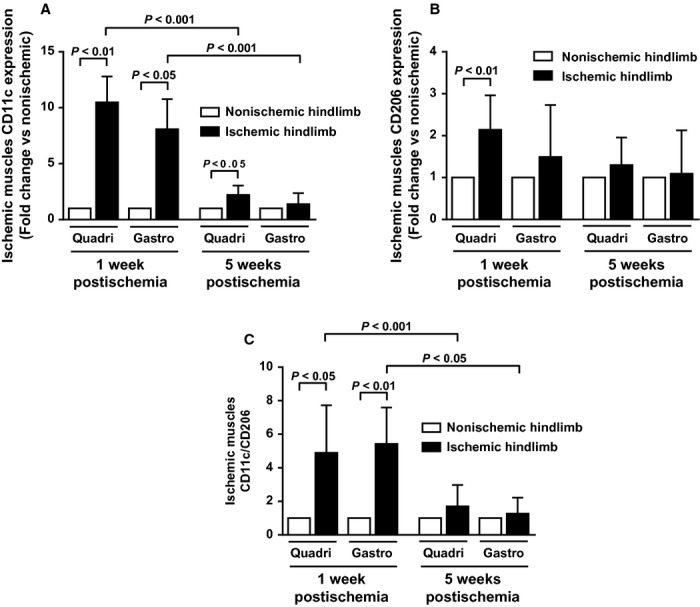
Hindlimb muscles macrophage phenotype (pro‐inflammatory M1 vs. anti‐inflammatory M2 macrophages) at early and later stages of peripheral ischemia. (A) mRNA expression of CD11c (M1 marker), and (B) mRNA expression of CD206 (M2 marker) in ischemic quadriceps (proximal to ischemia) and gastrocnemius (distal to ischemia) muscles as measured by real‐time PCR. Results are expressed as fold change in expression over respective contralateral nonischemic muscles, set at 1. (C) Ratio of CD11c to CD206 (*n* = 7–10 animals per time point).

Macrophage phenotype was characterized by examining expression of specific pro‐inflammatory M1 (CD11c) and anti‐inflammatory M2 (CD206) macrophage markers using real‐time PCR. At early stage of ischemia, CD11c mRNA expression was significantly upregulated both in ischemic quadriceps (10.5‐fold, *P* < 0.01), and gastrocnemius muscles (8.1‐fold, *P* < 0.05) (Fig. 6A). At later stage, CD11c mRNA expression remained significantly upregulated exclusively in ischemic quadriceps muscle (2.2‐fold, *P* < 0.05) although values were significantly lower than those observed at 1 week (*P* < 0.001; Fig. 6A). During early phase of ischemia, CD206 mRNA expression was significantly upregulated (2.1‐fold) in ischemic quadriceps muscle only (*P* < 0.01; Fig. 6B). Based on these results, calculation of CD11c to CD206 ratio (index of M1/M2 macrophage balance) resulted significantly higher both in quadriceps (4.9‐fold, *P* < 0.05), and gastrocnemius (5.4‐fold, *P* < 0.01) ischemic muscles, compared to nonischemic ones, during early stage of limb ischemia. This increase was no longer significant at later stage (Fig. 6C).

### Phenotype of muscle T cells in early and late stages of limb ischemia

**Figure 7.**
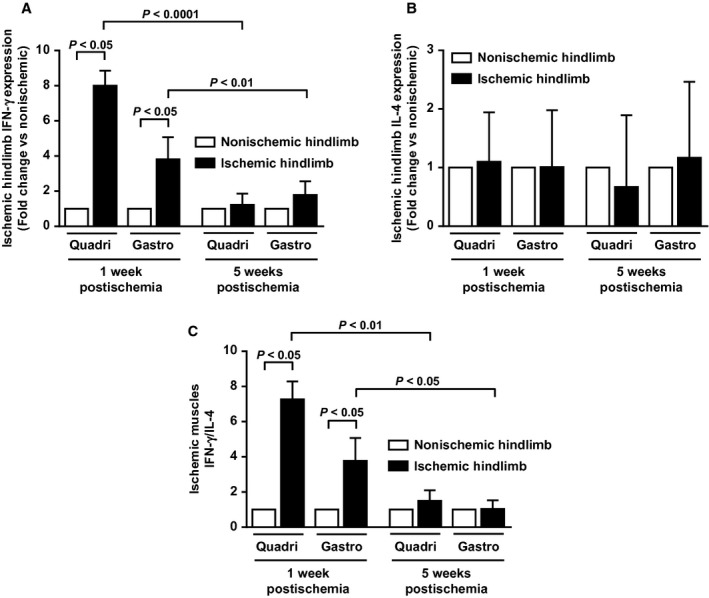
Hindlimb muscles T‐cell phenotype (pro‐inflammatory Th1 cells vs. anti‐inflammatory Th2 cells) at early and later stages of peripheral ischemia. (A) mRNA expression of the cytokine IFN‐*γ* (pro‐inflammatory Th1 marker), and (B) mRNA expression of IL‐4 (anti‐inflammatory Th2 marker) in ischemic quadriceps (proximal to ischemia) and gastrocnemius (distal to ischemia) as measured by real‐time PCR. Results are expressed as fold change in expression over respective contralateral nonischemic muscles, set at 1. (C), Ratio of IFN‐*γ* to IL‐4 (*n* = 4–9 animals per time point).

**Figure 8.**
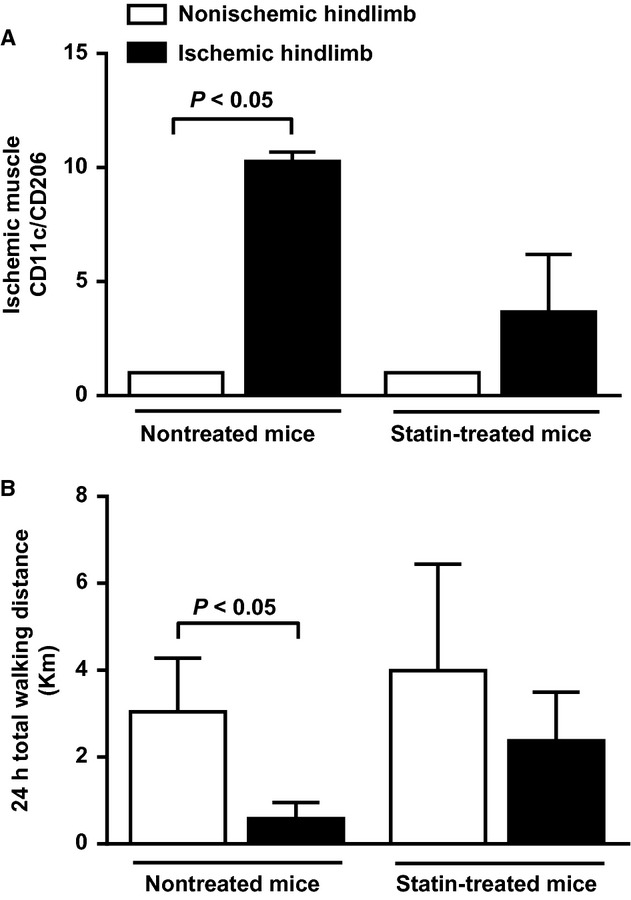
Effect of atorvastatin on hindlimb muscles macrophage phenotype (pro‐inflammatory M1 vs. anti‐inflammatory M2 macrophages) and walking ability of mice at early stage of peripheral ischemia. (A) Ratio of CD11c to CD206 (*n* = 3 animals per group), and (B) Quantification of 24 h total walking distance (24hTWD) using a 24 h voluntary running wheel test (*n* = 3 animals per group).

Phenotype of T cells (pro‐inflammatory Th1 cells vs. anti‐inflammatory Th2 cells) in hindlimb muscles was also evaluated. As shown in Figure 8A, IFN‐*γ* mRNA expression (pro‐inflammatory Th1 marker), was significantly upregulated in early ischemic phase both in quadriceps (8.0‐fold, *P* < 0.05 vs. nonischemic one) and gastrocnemius (3.8‐fold, *P* < 0.05 vs. nonischemic one) muscles. However, this upregulation was no longer present in later ischemic stage (Fig. 7A). IL‐4 mRNA expression (anti‐inflammatory Th2 marker) was not significantly modulated by ischemia, both at early and late phases (Fig. 7B). As a consequence, IFN‐*γ*/IL‐4 ratio (index of Th1/Th2 cell balance) resulted significantly higher in ischemic quadriceps (7.3‐fold), and gastrocnemius (3.8‐fold) muscles during early phase of ischemia (*P* < 0.05 vs. respective nonischemic muscles; Fig. 7C).

### Effect of muscle inflammation inhibition on walking ability of mice in early stage of limb ischemia

Markers of M1/M2 macrophage balance were determined in quadriceps muscle 1 week postischemia in mice treated with or without atorvastatin. As shown in Figure 8A, while the ratio of CD11c to CD206 was significantly higher in ischemic quadriceps muscle than in nonischemic one in control nontreated mice (*P* < 0.05), no significant difference was observed in atorvastatin‐treated mice. Interestingly, atorvastatin treatment prevented the significant decrease in 24hTWD observed in control mice (*P* < 0.05) (Fig. 8B).

## Discussion

Results from this study show that in a mouse model of PAD, different muscular adaptive mechanisms take place in response to early and late stages of ischemia. During early phase, muscle glucose uptake raises significantly while decreasing at later phases. Local inflammatory reactions take place with significant macrophage and polarization of T cells toward a pro‐inflammatory phenotype during early phase. This inflammatory imbalance is, however, restored at later phases.

It is known that abnormal ischemic limb hemodynamic status (reduced limb oxygenation and perfusion) does not completely explain functional limitations experienced by patients with PAD (Szuba et al. 2006; Pipinos et al. 2007; Anderson et al. 2009). Our mouse model closely reproduces this human process. Indeed, walking capacity remains profoundly impaired even though limb perfusion and oxygenation tends to significantly increase with time (though remaining inferior to the pre‐ischemic situation). Along the same line, muscle arteriolar density tends to increase. This corroborates the hypothesis that additional intrinsic ischemic muscle factors, independent of hemodynamic ones, are likely to play a role in PAD pathophysiology.

Plasma‐derived glucose importantly contributes to muscle energetic fueling. PET scan, using ^18^FDG glucose tracer, is a well‐established approach to measure in vivo skeletal muscle glycolytic activity (Kelley et al. 2001; Gondoh et al. 2009). It allowed us to demonstrate, for the first time in a mouse model of PAD, a significantly increased glucose uptake in resting ischemic mouse muscles during early phase of ischemia. Modulation of glucose uptake during early ischemia was also documented in a porcine model of myocardial infarction (Lautamaki et al. 2009). In this study, ^18^FDG PET also revealed increased glucose uptake in the hypoperfused infarcted area early after myocardial infarction. Contrary to the early phase situation, during the later phase of ischemia, we observed a significantly decreased glucose uptake. This result is in accordance with a recent study showing decreased calf muscle glucose uptake in chronic PAD patients with IC compared to healthy control subjects (Pande et al. 2011). Interestingly, this metabolic abnormality characterizing later phases of peripheral ischemia in our model relates to impaired walking ability, suggesting that decreased muscle glucose uptake may predict exercise limitation. Taken together, these findings suggest that transient initial increase in muscle glucose uptake allows maintaining basal muscle viability despite significantly reduced limb perfusion but that at later stages failure to maintain a sustained muscle glucose metabolism impairs ameliorating walking performances despite increased limb perfusion. Future investigations are needed to determine molecular mechanisms responsible for glucose uptake modulation occurring in peripheral ischemia.

Systemic inflammation has been shown to play a role in the pathophysiology of PAD. Indeed, previous works showed a strong relationship between elevated serum or plasma levels of various inflammatory markers and PAD claudication severity (McDermott et al. 2008; Brevetti et al. 2010). The implication of inflammation at the local level (i.e., muscle) remains, however, poorly investigated. Previous works have demonstrated presence of M1 and M2‐polarized macrophages as well as CD4 Th1 and Th2 cells in atherosclerotic plaques. Additionally, growing evidence strongly suggests that modulation of the M1/M2 and/or the Th1/Th2 balance affects pathogenesis, evolution, and complications of atherosclerosis (Mantovani et al. 2009; Ketelhuth and Hansson 2011; Hoeksema et al. 2012). Although macrophage and T‐cell balance plays a major role in atherosclerosis, few experimental data are available as yet to substantiate their role in PAD. In the present work, phenotypic analysis of muscle infiltrating M1, M2 macrophages and Th1, Th2 cells revealed that early ischemia is accompanied by macrophage and T‐cell balance shift toward a pro‐inflammatory state. This proinflammatory phenotypic switch returns to neutral state at later stages of ischemia. Therefore, pro‐inflammatory cells may be critical players in the initial phase of ischemic events, and may contribute to impaired walking capacity. To test this hypothesis, mice were treated with a statin to selectively inhibit muscular inflammation, especially pro‐inflammatory M1 activation state. The rational for the use of statin as a therapeutic agent to inhibit macrophage polarization has been demonstrated recently (Li et al. 2013; van der Meij et al. 2013). Results show that M1/M2 shift inhibition at early stage of limb ischemia prevented impaired mice walking ability, thereby demonstrating that pro‐inflammatory muscular state plays a critical role in PAD‐related impaired walking ability in our mouse model. ^18^FDG used in PET imaging is uptaken by macrophages (Joshi et al. 2011). Thus, PET scan results showing increased muscle glucose uptake in early ischemic phase may also reflect increased number of infiltrating pro‐inflammatory macrophages. Rapid local muscle inflammation in response to ischemia may, therefore, be a transient deleterious mechanism contributing to walking ability impairment. With time, compensatory increased limb perfusion will provide sufficient basal state muscle nutriment though not enough to guarantee appropriate energy fueling during functional activity (i.e., walking).

To gain insights into skeletal muscle energy metabolism, in response to early and late stages of ischemia, index of energetic state (PCr/*γ*‐ATP and PCr/Pi ratios) was measured using ^31^P‐MRS. ^31^P‐MRS is at present the only available technique permitting noninvasive in vivo measurement of major phosphorylated compounds involved in muscle energy metabolism: that is, ATP, and PCr (Boesch 2007). ATP is a substrate for all energy‐consuming cellular reactions, as its hydrolysis provides free energy (Ten Hove and Neubauer 2007). PCr acts as an energy buffer and serves as an energy transport molecule in the creatine kinase/PCr energy shuttle (Ten Hove and Neubauer 2007). No differences in resting ischemic muscle energy state were observed in our mouse model, both in early and late phases of limb ischemia, relatively to nonischemic contralateral limb. On the other hand, Boring et al. (2013) recently showed a drop of PCr and ATP levels 1 week after femoral artery ligation and excision in C57BL/6 wild‐type mice, compared to those before femoral ligation. In our ^31^P‐MRS experiments, a drop in the PCr/*γ*‐ATP concentration after iliac artery ligation and a significant increase in PCr/*γ*‐ATP and PCr/Pi ratios between early and late ischemic stages were observed not only in the ischemic hindlimb but also in the contralateral nonischemic one used as internal control for each mouse (data not shown). This phenomenon may be due to a systemic response following unilateral arterial occlusion, as previously reported (Yan et al. 2009), and may have interfered with local metabolite changes in ischemic limb in this study. However, clinical studies also reported no significant differences in resting muscle metabolism between PAD patients and control subjects (Pedersen et al. 2009). Similar concentrations of high‐energy phosphate metabolites have also been reported under resting conditions in patients with type 2 diabetes and hypercholesterolemia, two majors CV risk factors for PAD, as compared to corresponding control healthy subjects (Laaksonen et al. 1996; Scheuermann‐Freestone et al. 2003). Taken together, these data indicate that energy reserve is unchanged in resting skeletal muscle compared to nonischemic muscle at any stage of peripheral ischemia.

In conclusion, results presented herein provide novel insights into the intrinsic adaptive mechanisms taking place in early and late phases of muscle ischemia. These ischemia‐associated changes in skeletal muscles may contribute to PAD‐related walking ability impairment.

### Clinical perspective

PAD is a common disorder limiting patient's walking ability. To date, no pharmacological therapy for PAD exists. Only a better understanding of biological mechanisms underlying PAD may help develop new therapeutic strategies aiming at improving patient function and quality of life. This study indicates that both pro‐inflammatory T cells and macrophages are implicated in early ischemia whereas late ischemia is associated with impaired muscle glucose uptake in a mouse model of PAD. Hence, treatment aiming at preventing modulation of muscle macrophage and T cells toward a pro‐inflammatory phenotype in early disease stage and/or impaired muscle glucose uptake during late disease stage might improve clinical symptoms of patients with PAD.

## Acknowledgments

We thank Pascal Laurant (University of Avignon, Avignon, France) for lending us the rodent treadmill.

## Conflict of Interest

None declared.

## References

[b1] Anderson J. D. Epstein F. H. Meyer C. H. Hagspiel K. D. Wang H. Berr S. S. 2009 Multifactorial determinants of functional capacity in peripheral arterial disease: uncoupling of calf muscle perfusion and metabolism. J. Am. Coll. Cardiol.; 54:628-635 19660694 10.1016/j.jacc.2009.01.080 PMC2768062

[b2] Boesch C. 2007 Musculoskeletal spectroscopy. J. Magn. Reson. Imaging; 25:321-338 17260389 10.1002/jmri.20806

[b3] Boring Y. C. Flogel U. Jacoby C. Heil M. Schaper W. Schrader J. 2013 Lack of ecto‐5′‐nucleotidase (CD73) promotes arteriogenesis. Cardiovasc. Res.; 97:88-96 22977005 10.1093/cvr/cvs286

[b4] Brevetti G. Giugliano G. Brevetti L. Hiatt W. R. 2010 Inflammation in peripheral artery disease. Circulation; 122:1862-1875 21041698 10.1161/CIRCULATIONAHA.109.918417

[b6] Gondoh Y. Tashiro M. Itoh M. Masud M. M. Sensui H. Watanuki S. 2009 Evaluation of individual skeletal muscle activity by glucose uptake during pedaling exercise at different workloads using positron emission tomography. J. Appl. Physiol.; 107:599-604 19541734 10.1152/japplphysiol.90821.2008

[b7] Gruetter R. Tkac I. 2000 Field mapping without reference scan using asymmetric echo‐planar techniques. Magn. Reson. Med.; 43:319-323 10680699 10.1002/(sici)1522-2594(200002)43:2<319::aid-mrm22>3.0.co;2-1

[b8] Hansson G. K. Hermansson A. 2011 The immune system in atherosclerosis. Nat. Immunol.; 12:204-212 21321594 10.1038/ni.2001

[b9] Hirsch A. T. Haskal Z. J. Hertzer N. R. Bakal C. W. Creager M. A. Halperin J. L. 2006 ACC/AHA 2005 Practice Guidelines for the management of patients with peripheral arterial disease (lower extremity, renal, mesenteric, and abdominal aortic): a collaborative report from the American Association for Vascular Surgery/Society for Vascular Surgery, Society for Cardiovascular Angiography and Interventions, Society for Vascular Medicine and Biology, Society of Interventional Radiology, and the ACC/AHA Task Force on Practice Guidelines (Writing Committee to Develop Guidelines for the Management of Patients With Peripheral Arterial Disease): endorsed by the American Association of Cardiovascular and Pulmonary Rehabilitation; National Heart, Lung, and Blood Institute; Society for Vascular Nursing; TransAtlantic Inter‐Society Consensus; and Vascular Disease Foundation. Circulation; 113:e463-e654 16549646 10.1161/CIRCULATIONAHA.106.174526

[b10] Hoeksema M. A. Stoger J. L. de Winther M. P. 2012 Molecular pathways regulating macrophage polarization: implications for atherosclerosis. Curr. Atheroscler. Rep.; 14:254-263 22407286 10.1007/s11883-012-0240-5 PMC3348484

[b11] Joshi F. Rosenbaum D. Bordes S. Rudd J. H. 2011 Vascular imaging with positron emission tomography. J. Intern. Med.; 270:99-109 21518037 10.1111/j.1365-2796.2011.02392.x

[b12] Kelley D. E. Price J. C. Cobelli C. 2001 Assessing skeletal muscle glucose metabolism with positron emission tomography. IUBMB Life; 52:279-284 11895076 10.1080/152165401317291129

[b13] Ketelhuth D. F. Hansson G. K. 2011 Cellular immunity, low‐density lipoprotein and atherosclerosis: break of tolerance in the artery wall. Thromb. Haemost.; 106:779-786 21979058 10.1160/TH11-05-0321

[b14] Laaksonen R. Jokelainen K. Laakso J. Sahi T. Harkonen M. Tikkanen M. J. 1996 The effect of simvastatin treatment on natural antioxidants in low‐density lipoproteins and high‐energy phosphates and ubiquinone in skeletal muscle. Am. J. Cardiol.; 77:851-854 8623738 10.1016/S0002-9149(97)89180-1

[b15] Lautamaki R. Schuleri K. H. Sasano T. Javadi M. S. Youssef A. Merrill J. 2009 Integration of infarct size, tissue perfusion, and metabolism by hybrid cardiac positron emission tomography/computed tomography: evaluation in a porcine model of myocardial infarction. Circ. Cardiovasc. Imaging; 2:299-305 19808610 10.1161/CIRCIMAGING.108.846253

[b16] Li Q. Z. Sun J. Han J. J. Qian Z. J. 2013 Anti‐inflammation of simvastatin by polarization of murine macrophages from M1 phenotype to M2 phenotype. Zhonghua Yi Xue Za Zhi; 93:2071-2074 24169290

[b17] Livak K. J. Schmittgen T. D. 2001 Analysis of relative gene expression data using real‐time quantitative PCR and the 2(‐Delta Delta C(T)) Method. Methods; 25:402-408 11846609 10.1006/meth.2001.1262

[b18] Mantovani A. Garlanda C. Locati M. 2009 Macrophage diversity and polarization in atherosclerosis: a question of balance. Arterioscler. Thromb. Vasc. Biol.; 29:1419-1423 19696407 10.1161/ATVBAHA.108.180497

[b19] Mazzolai L. Duchosal M. A. Korber M. Bouzourene K. Aubert J. F. Hao H. 2004 Endogenous angiotensin II induces atherosclerotic plaque vulnerability and elicits a Th1 response in ApoE−/− mice. Hypertension; 44:277-282 15302839 10.1161/01.HYP.0000140269.55873.7b

[b20] McDermott M. M. Liu K. Ferrucci L. Tian L. Guralnik J. M. Green D. 2008 Circulating blood markers and functional impairment in peripheral arterial disease. J. Am. Geriatr. Soc.; 56:1504-1510 18662216 10.1111/j.1532-5415.2008.01797.x PMC2658758

[b21] van der Meij E. Koning G. G. Vriens P. W. Peeters M. F. Meijer C. A. Kortekaas K. E. 2013 A clinical evaluation of statin pleiotropy: statins selectively and dose‐dependently reduce vascular inflammation. PLoS One; 8:e53882 23349755 10.1371/journal.pone.0053882 PMC3551939

[b22] Mlynarik V. Gambarota G. Frenkel H. Gruetter R. 2006 Localized short‐echo‐time proton MR spectroscopy with full signal‐intensity acquisition. Magn. Reson. Med.; 56:965-970 16991116 10.1002/mrm.21043

[b23] Norgren L. Hiatt W. R. Dormandy J. A. Nehler M. R. Harris K. A. Fowkes F. G. 2007 Inter‐society consensus for the management of peripheral arterial disease (TASC II). J. Vasc. Surg.; 45Suppl. S:S5-S67 17223489 10.1016/j.jvs.2006.12.037

[b24] Olin J. W. Allie D. E. Belkin M. Bonow R. O. Casey D. E. Jr. Creager M. A. 2010 ACCF/AHA/ACR/SCAI/SIR/SVM/SVN/SVS 2010 performance measures for adults with peripheral artery disease: a report of the American College of Cardiology Foundation/American Heart Association Task Force on performance measures, the American College of Radiology, the Society for Cardiac Angiography and Interventions, the Society for Interventional Radiology, the Society for Vascular Medicine, the Society for Vascular Nursing, and the Society for Vascular Surgery (Writing Committee to Develop Clinical Performance Measures for Peripheral Artery Disease). Circulation; 122:2583-2618 21126978 10.1161/CIR.0b013e3182031a3c

[b25] Pande R. L. Park M. A. Perlstein T. S. Desai A. S. Doyle J. Navarrete N. 2011 Impaired skeletal muscle glucose uptake by [18F]fluorodeoxyglucose‐positron emission tomography in patients with peripheral artery disease and intermittent claudication. Arterioscler. Thromb. Vasc. Biol.; 31:190-196 21051665 10.1161/ATVBAHA.110.217687

[b26] Pedersen B. L. Baekgaard N. Quistorff B. 2009 Muscle mitochondrial function in patients with type 2 diabetes mellitus and peripheral arterial disease: implications in vascular surgery. Eur. J. Vasc. Endovasc. Surg.; 38:356-364 19524462 10.1016/j.ejvs.2009.04.014

[b27] Pipinos I. I. Judge A. R. Selsby J. T. Zhu Z. Swanson S. A. Nella A. A. 2007 The myopathy of peripheral arterial occlusive disease: part 1. Functional and histomorphological changes and evidence for mitochondrial dysfunction. Vasc. Endovascular Surg.; 41:481-489 18166628 10.1177/1538574407311106

[b28] Pipinos I. I. Judge A. R. Selsby J. T. Zhu Z. Swanson S. A. Nella A. A. 2008 The myopathy of peripheral arterial occlusive disease: part 2. Oxidative stress, neuropathy, and shift in muscle fiber type. Vasc. Endovascular Surg.; 42:101-112 18390972 10.1177/1538574408315995 PMC12282609

[b29] Scheuermann‐Freestone M. Madsen P. L. Manners D. Blamire A. M. Buckingham R. E. Styles P. 2003 Abnormal cardiac and skeletal muscle energy metabolism in patients with type 2 diabetes. Circulation; 107:3040-3046 12810608 10.1161/01.CIR.0000072789.89096.10

[b30] Selivanov V. V. P. Y. Cadorette J. Rodrigue S. Lecomte R. 2000 Detector response models for statistical iterative image reconstruction in high resolution PET. IEEE Trans. Nucl. Sci.; 47:1168-1175

[b31] Seyer P. Vallois D. Poitry‐Yamate C. Schutz F. Metref S. Tarussio D. 2013 Hepatic glucose sensing is required to preserve beta cell glucose competence. J. Clin. Invest.; 123:1662-1676 23549084 10.1172/JCI65538 PMC3613916

[b32] Silvestre J. S. Mallat Z. Tedgui A. Levy B. I. 2008 Post‐ischaemic neovascularization and inflammation. Cardiovasc. Res.; 78:242-249 18252762 10.1093/cvr/cvn027

[b33] Szuba A. Oka R. K. Harada R. Cooke J. P. 2006 Limb hemodynamics are not predictive of functional capacity in patients with PAD. Vasc. Med.; 11:155-163 17288121 10.1177/1358863x06074828

[b34] Ten Hove M. Neubauer S. 2007 MR spectroscopy in heart failure–clinical and experimental findings. Heart Fail. Rev.; 12:48-57 17333358 10.1007/s10741-007-9003-8

[b35] Vanhamme L. van den Boogaart A. Van Huffel S. 1997 Improved method for accurate and efficient quantification of MRS data with use of prior knowledge. J. Magn. Reson.; 129:35-43 9405214 10.1006/jmre.1997.1244

[b36] Wang J. A. Chen W. A. Wang Y. Zhang S. Bi H. Hong B. 2011 Statins exert differential effects on angiotensin II‐induced atherosclerosis, but no benefit for abdominal aortic aneurysms. Atherosclerosis; 217:90-96 21481872 10.1016/j.atherosclerosis.2011.03.005

[b37] Yan J. Tie G. Park B. Yan Y. Nowicki P. T. Messina L. M. 2009 Recovery from hind limb ischemia is less effective in type 2 than in type 1 diabetic mice: roles of endothelial nitric oxide synthase and endothelial progenitor cells. J. Vasc. Surg.; 50:1412-1422 19837544 10.1016/j.jvs.2009.08.007 PMC2797079

